# PANS Case Report. Assessment and management implications for a Liaison Child Psychiatry Program

**DOI:** 10.1192/j.eurpsy.2023.1531

**Published:** 2023-07-19

**Authors:** M. R. Perez Moreno, M. Huete Naval

**Affiliations:** 1 Instituto de psiquiatría y salud mental, hospital clínico san carlos, Madrid, Spain

## Abstract

**Introduction:**

Pediatric acute-onset neuropsychiatric syndrome (PANS) was described in 2010 not related to streptococcus infection (as PANDAS is), and with a clinically distinct presentation, defined as: I) Abrupt, dramatic onset of obsessive-compulsive disorder or severely restricted food intake; II) Concurrent presence of additional neuropsychiatric symptoms; III) Symptoms are not better explained by a known neurologic or medical disorder.

**Objectives:**

To describe the clinical features in a scholar boy who suffered an abrupt obsessive-compulsive disorder and highlight the need of an specific medical and psychiatric assessment and management from a multidisciplinary perspective.

**Methods:**

Clinical case: A 7-year-old boy brought to the emergency department due to his repetitive and hyperactive behavior. After the admission in the hospital a clinical history was identified with PANS diagnostic criteria. He presented repetitive language and ritualized behavior, emotional lability and hyperactivity that has begun in an abrupt manner in the last 5 days. Family history, medical history and physical examination, infectious disease evaluation, neurological assessment and child psychiatric assessment were carried out during hospitalization. Coordination between neuropediatric consultant and child psychiatry was necessary.

**Results:**

Combinated treatment, psychofarmacologic and psychotherapeutic, was effective and the symptoms disapeared gradually in about three months.

**Conclusions:**

In all school-age child presenting with abrupt obsessive-compulsive disorder or eating disorders a possible link to PANS should be evaluated and rule out. It is important a Liaison Child Psychiatry program for a complete multidisciplinary evaluation and management of these patients.

**Disclosure of Interest:**

None Declared

